# Quantitative Screening of Cervical Cancers for Low-Resource Settings: Pilot Study of Smartphone-Based Endoscopic Visual Inspection After Acetic Acid Using Machine Learning Techniques

**DOI:** 10.2196/16467

**Published:** 2020-03-11

**Authors:** Jung Kweon Bae, Hyun-Jin Roh, Joon S You, Kyungbin Kim, Yujin Ahn, Sanzhar Askaruly, Kibeom Park, Hyunmo Yang, Gil-Jin Jang, Kyung Hyun Moon, Woonggyu Jung

**Affiliations:** 1 Department of Biomedical Engineering Ulsan National Institute of Science and Technology Ulsan Republic of Korea; 2 Department of Obstetrics and Gynaecology University of Ulsan College of Medicine Ulsan University Hospital Ulsan Republic of Korea; 3 Department of Pathology Ulsan University Hospital Ulsan Republic of Korea; 4 School of Electronics Engineering Kyungpook National University Daegu Republic of Korea; 5 Department of Urology University of Ulsan College of Medicine Ulsan University Hospital Ulsan Republic of Korea

**Keywords:** smartphone-based endoscope, smartphone VIA, machine learning, cervical cancer screening, low-resource settings

## Abstract

**Background:**

Approximately 90% of global cervical cancer (CC) is mostly found in low- and middle-income countries. In most cases, CC can be detected early through routine screening programs, including a cytology-based test. However, it is logistically difficult to offer this program in low-resource settings due to limited resources and infrastructure, and few trained experts. A visual inspection following the application of acetic acid (VIA) has been widely promoted and is routinely recommended as a viable form of CC screening in resource-constrained countries. Digital images of the cervix have been acquired during VIA procedure with better quality assurance and visualization, leading to higher diagnostic accuracy and reduction of the variability of detection rate. However, a colposcope is bulky, expensive, electricity-dependent, and needs routine maintenance, and to confirm the grade of abnormality through its images, a specialist must be present. Recently, smartphone-based imaging systems have made a significant impact on the practice of medicine by offering a cost-effective, rapid, and noninvasive method of evaluation. Furthermore, computer-aided analyses, including image processing–based methods and machine learning techniques, have also shown great potential for a high impact on medicinal evaluations.

**Objective:**

In this study, we demonstrate a new quantitative CC screening technique and implement a machine learning algorithm for smartphone-based endoscopic VIA. We also evaluated the diagnostic performance and practicability of the approach based on the results compared to the gold standard and from physicians’ interpretation.

**Methods:**

A smartphone-based endoscope system was developed and applied to the VIA screening. A total of 20 patients were recruited for this study to evaluate the system. Overall, five were healthy, and 15 were patients who had shown a low to high grade of cervical intraepithelial neoplasia (CIN) from both colposcopy and cytology tests. Endoscopic VIA images were obtained before a loop electrosurgical excision procedure for patients with abnormal tissues, and their histology tissues were collected. Endoscopic VIA images were assessed by four expert physicians relative to the gold standard of histopathology. Also, VIA features were extracted from multiple steps of image processing techniques to find the differences between abnormal (CIN2+) and normal (≤CIN1). By using the extracted features, the performance of different machine learning classifiers, such as k-nearest neighbors (KNN), support vector machine, and decision tree (DT), were compared to find the best algorithm for VIA. After determining the best performing classifying model, it was used to evaluate the screening performance of VIA.

**Results:**

An average accuracy of 78%, with a Cohen kappa of 0.571, was observed for the evaluation of the system by four physicians. Through image processing, 240 sliced images were obtained from the cervicogram at each clock position, and five features of VIA were extracted. Among the three models, KNN showed the best performance for finding VIA within holdout 10-fold cross-validation, with an accuracy of 78.3%, area under the curve of 0.807, a specificity of 80.3%, and a sensitivity of 75.0%, respectively. The trained model performed using an unprovided data set resulted in an accuracy of 80.8%, specificity of 84.1%, and sensitivity of 71.9%. Predictions were visualized with intuitive color labels, indicating the normal/abnormal tissue using a circular clock-type segmentation. Calculating the overlapped abnormal tissues between the gold standard and predicted value, the KNN model overperformed the average assessments of physicians for finding VIA.

**Conclusions:**

We explored the potential of the smartphone-based endoscopic VIA as an evaluation technique and used the cervicogram to evaluate normal/abnormal tissue using machine learning techniques. The results of this study demonstrate its potential as a screening tool in low-resource settings.

## Introduction

According to the International Agency for Research on Cancer and GLOBOCAN 2018, cervical cancer (CC) is the fourth most frequent cancer in women worldwide [1], and approximately 90% of the global cervical cancer deaths in 2015 occurred in low- and middle-income countries [2,3]. Although CC is regarded as a highly preventable and curable cancer, it is still one of the leading causes of mortality in low-resource settings and developing countries due to their lack of sustainable screening programs and limited infrastructure [3-5]. CC can be readily managed when it is found in the precancerous stages through routine screening methods, such as a cytology-based test. The most popular and affordable method for CC screening in low-resource countries is the use of visual inspection with acetic acid (VIA). Since VIA offers relatively simple, cost-effective visual feedback, it can even provide treatment on the same day of a screening visit [4-7]. In VIA, the topical application of 4-5% acetic acid to the cervix transforms abnormal squamous epithelium to a dense white color, while normal epithelium presents as a light pink color. Despite its simplicity, VIA provides sufficient sensitivity and specificity to identify the cancerous lesion; thus, it has been widely promoted and recommended as an alternative to the conventional cytology test (ie, the Pap smear) [4-8]. Nonetheless, visual inspection methods have been found to be subjective and the range of diagnostic performance varies widely, with significantly better results obtained by physicians than by nurses [8]. Unfortunately, in many developing countries, trained physicians who can interpret VIA correctly may not be readily available [5,7,8].

Digital images of the cervix after application of acetic acid, or digital cervicography, have been significantly important for improving quality control. It is a very efficient way of minimizing interpreters’ subjectivity by capturing higher resolution images for post-screening analysis [9-13]. Moreover, digital images can be transmitted or shared through the internet with long-distance experts, thus closing the gap in human resources [13]. Recent advances in smartphone technologies have opened new possibilities for cervical screening in low-resource settings, thus overcoming the limitations of colposcopy, including the device’s bulkiness, high-cost, electricity dependency, and constant maintenance need [14-20].

The smartphone is a highly integrated platform that includes various functionalities, easy accessibility, a user-friendly interface, ubiquitous internet, and communication technologies [21]. The high-definition camera in a smartphone has especially made an impact on the practice of medicine by offering cost-effective, rapid, and noninvasive imaging capabilities [21-25]. Smartphone-based cervical screening has been proven feasible and validated for quality assurance in low resource settings [14-20]. Smartphone-based digital visual inspection following application of acetic acid has been demonstrated for higher diagnostic accuracy and reduction of the variability of detection rate. Although digital images are very effective in various ways [5], implementation of remote expert consultation is still challenging due to the lack of reliable broadband connections in remote areas [19].

On the other hand, automated interpretation of data and classification of cervical images for instant diagnostic conclusions will enable on-site treatments to be delivered without delays [26-31]. To date, various image processing and interpretation methods have been successfully applied to VIA using such features as aceto-whitening, blood vessel formation, and texture of the surface [29-31]. Previous works have shown that automated classification of VIA can perform as well as experts’ qualitative assessment of colposcopic images [26-28]. Also, auxiliary processing methods, such as elimination of speculum reflection and determination of the region of interest (ROI), can further improve the overall performance of image processing outcomes [31]. Automated quantification of VIA based on modern image processing and machine learning techniques could be a very promising platform for cervical screening in low-resource settings. However, a fully automated diagnostic performance using smartphone-based cervical images has not been introduced, despite a clear need and potential.

In this study, we demonstrate a new quantitative CC screening technique by implementing a machine learning algorithm for smartphone-based endoscopic VIA. Our method can provide digital images as well as an automated diagnostic classification for comprehensive and intuitive feedback to a clinician. We have evaluated the diagnostic performance of the system through quantitative comparison to the gold standard of cytology and physicians’ interpretation of the digital images. This approach would extend cervical cancer screening to remote populations who do not have access to experienced colposcopists.

## Methods

### Smartphone-Based Endoscope System

**Figure 1:**
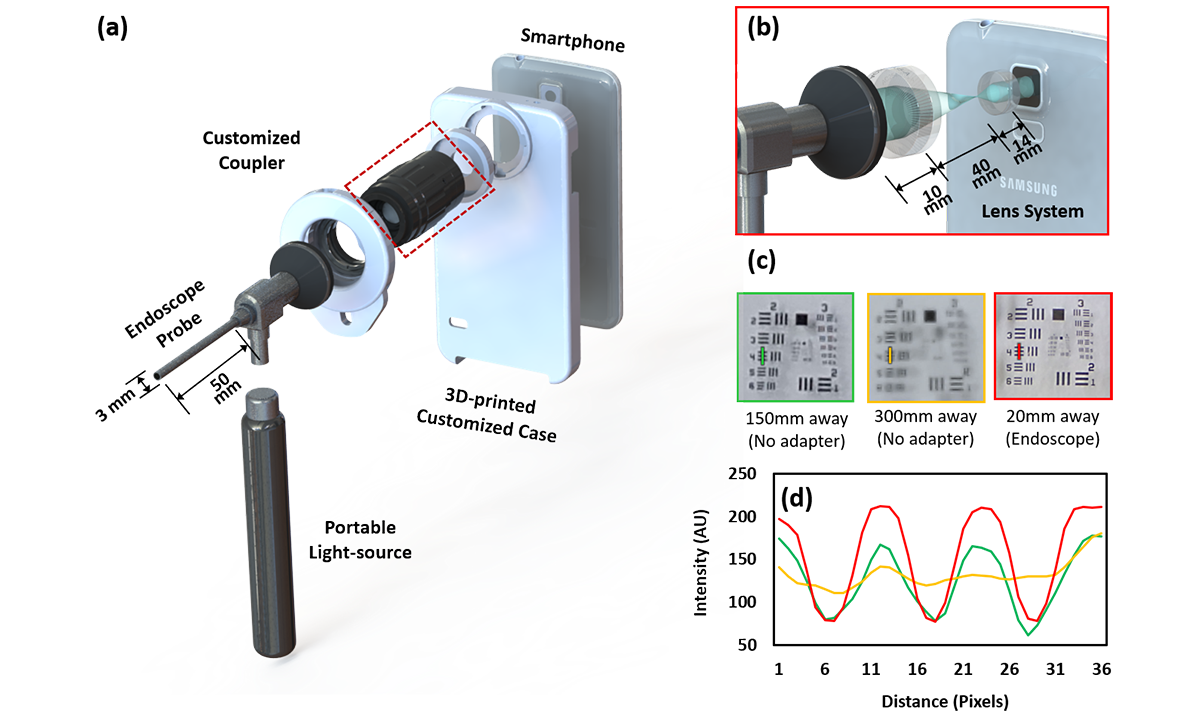
Schematic of the system. (a) 3D modeling of the smartphone-based endoscope system. (b) Optics of the customized zoom lens. (c) Images of the resolution target taken without any optics adapter and our system, respectively. (d) Group 2, element 4 from each image was described as a line plot. 3D: three-dimensional; AU: arbitrary unit.

We developed a miniaturized endoscope system by assembling an endoscopic probe and smartphone with customized ancillary components, as reported previously [25]. The smartphone-based endoscope system and its components are illustrated in Figure 1. The system is composed of 3 major components: (1) Customized coupler for universal attachment of endoscopic probes, generated by three-dimensional (3D) printer (Stratasys, Objet260 Connex2); (2) smartphone case, also generated by 3D printer; and (3) an optical adapter used for magnification, which is placed between the endoscopic probe and the smartphone camera, as shown in Figure 1, part (a). Incorporating achromatic and aspherized achromatic lenses that had 40-millimeter and 14-millimeter focal lengths (Figure 1, part [b]), respectively, we obtained approximately 4× optical magnification. The image can be further magnified up to approximately 12× with a smartphone’s digital zoom feature. A portable light source was attached to the illumination port of an endoscopic probe. Images were acquired with the home-built android application that features control functions such as compensation of the rotated images caused by the lens, camera controls, including zoom, ISO sensitivity, white balance, resolution size, and exposure adjustments, and the option to save files.

Optical elements of the adapter significantly improved the cervix image captured with a smartphone camera alone. Images of the central part of the Unites States air force (USAF) resolution target were captured with/without a smartphone endoscope (Figure 1, part [c]). The device was placed at 150 millimeters and 300 millimeters away from the target, where prior smartphone-based VIA [14,19] and routine colposcopy [32] are conducted. For the endoscope system, we placed the distal end of the probe at 20 millimeters away from the target, where the whole ectocervix was well defined in the field of view. As shown in Figure 1, part (d), smartphone-based endoscopy achieved the best resolution from a line plot representing Group 2, Element 4, from part (c), in the resolution target.

### Image Acquisition From Clinic

**Figure 2:**
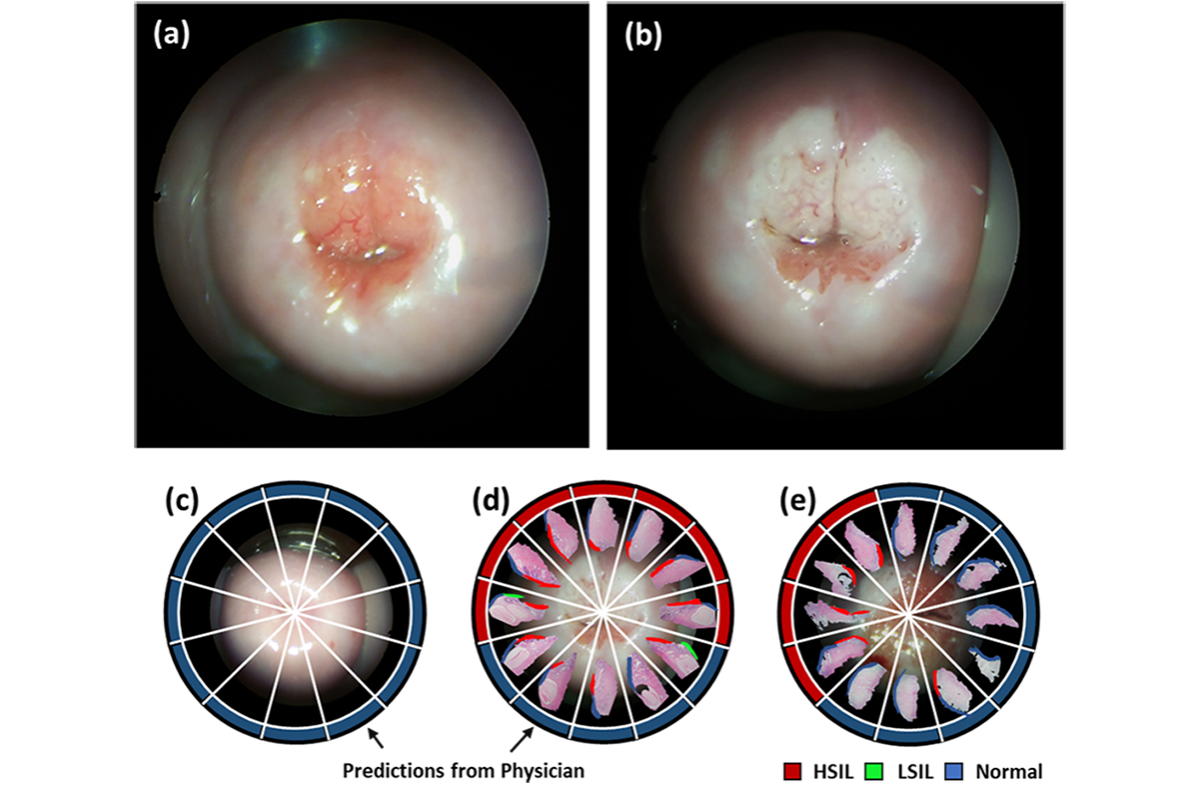
Smartphone-based endoscopic cervicogram. (a) Cervicogram of before acetic acid application. (b) Cervicogram of after acetic acid application. (c) VIA- patient (d-e) VIA+ patients with predictions from best among four physicians. Prediction labeled the precancerous regions with colors at each clock position. VIA: Visual inspection with acetic acid.

Following a protocol approved by the Ulsan University Hospital Institutional Review Board, we collected smartphone-based VIA images using an endoscope in human subjects. In Ulsan University Hospital, VIA was routinely performed to visualize the margin of the suspicious tissue before loop electrosurgical excision procedure (LEEP). Therefore, each imaging session was conducted before LEEP, in an operating room. A rigid endoscopic probe (Medstar, Otoscope, 0°, Ø4, and 50 mm length) was inserted inside a subject’s vagina where speculum had already been placed. Since the endoscope is thin, smartphone-based endoscopic VIA imaging was performed in a noninvasive and noncontact manner. All patients who underwent LEEP had already been potential candidates to have cervical intraepithelial neoplasia (CIN2+), which was determined by previous cytology-based tests and colposcopy. A typical procedure in this study took less than five minutes without causing undue burden on volunteers and delaying the treatment. First, one minute was used to take images before the application of the acetic acid, then the next 1-2 minutes was used to apply the 3-5% acetic acid, then another minute for waiting, and then the last minute was used to take VIA images. Figure 2, parts (a) and (b), show representative images of the smartphone-based endoscopic VIA. For patients who underwent LEEP, 12 tissue sections were collected at each clock position from the excised ectocervix. For this study, physicians labeled the CIN grades in colors, as shown Figure 2, parts (c)-(e). A total of 20 patients aged 20 years old or older participated in this study. Among them, five volunteers were normal (CIN1-), and 15 were confirmed abnormal (CIN1+) using the gold standard cytology test. Normal cervix status of the five subjects was verified as such by cytology test and colposcopy, so no LEEP was performed and no tissues were collected from them.

All captured images, including before and after application of the acetic acid, were sent to expert physicians for review. A total of four experts with professional experience, ranging from 12-20 years, participated and were kept blind to the results of the machine learning and the cytology. Physicians’ interpretations were based only on VIA features without any additional information given to them. During the image reviews, physicians labeled the directional information of the tissue region that contained suspicious abnormal features (Figure 2). In this study, tissues, including normal and CIN1, were considered to be normal, and CIN2+ was considered to be abnormal because only CIN2+ requires treatment [26].

### Preimage Processing

**Figure 3:**
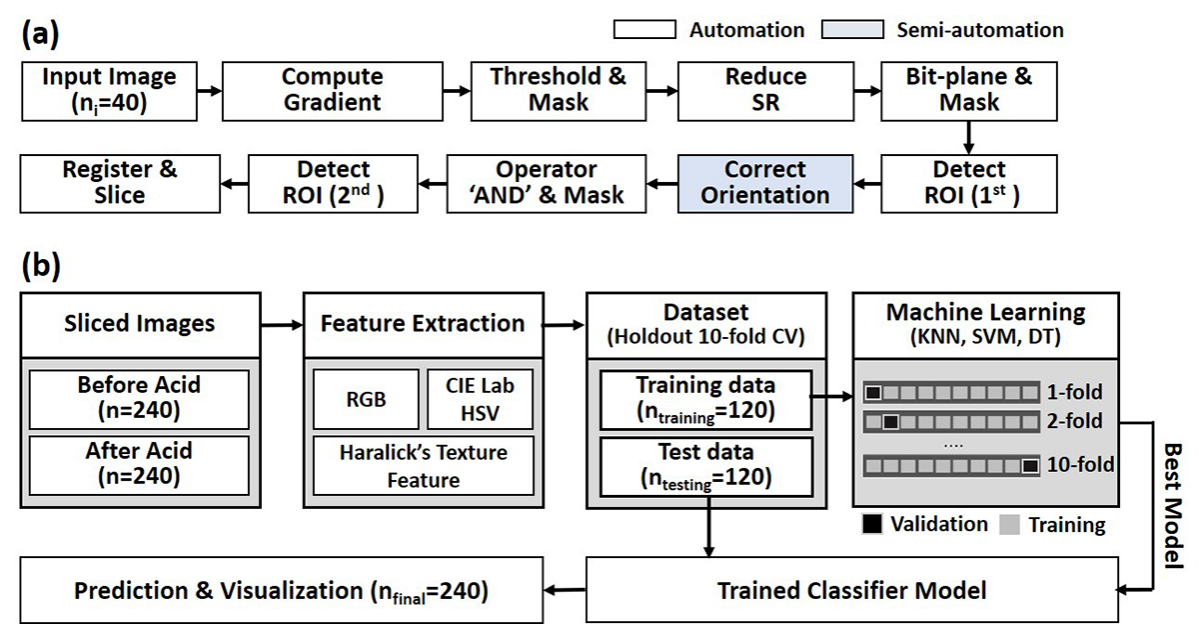
Block diagram of image processing and classification. (a) Preimage processing method, (b) Feature analysis and classification. SR: speculum reflection; ROI: region of interest; RGB: red, green, blue; CIE: Commission Internationale de l'Eclairage; HSV: hue, saturation, value; CV: cross-validation; KNN: K-nearest neighbor; SVM: supportive vector machine; DT: decision tree.

Images from smartphone-based endoscopic VIA contain unnecessary features, such as vaginal walls, speculum, and specular reflections of light, as commonly found in typical cervicograms [28,31]. As these features may affect overall classification accuracy, we performed multi-step image processing to reduce their influence on data analysis (See Figure 3a).

Next, ROI was defined to segment the major cervix region. The major cervix region in images both before and after the application of acetic acid contains high red-channel values [27]. For ROI detection, the red-channel image was transferred to grayscale and separated into multilevel binary images. Here, the 8-bit grayscale of the red-channel image was sliced into eight planes ranging from the least significant bit, 0, to the most significant bit, 7. Most of the seventh and eighth bit-planes represent the major ectocervix regions, so we used these features to generate the mask for ROI segmentation. Due to the slight differences of the cervix images between pre– and post–acetic acid application, we performed an ‘AND’ operator to segment the overlapping regions.

Before VIA features extraction and classification, image pairs for pre– and post–acetic acid application had to be properly registered. As shown in Multimedia Appendix 1, we manually provided three points as fiducials on each image to locate the center of the cervix (red dot) and both ends of the cervical os (2 black dots). Utilizing the center point, we correctly registered the center of the cervix for every image pair. We also drew a line penetrating the other two points and found the angle of the line from each image. We made this line horizontal by rotating images with respect to the given center point. Further, we cropped the images into 12 pieces, as sectioned in histology. In this image processing method, we used a total of 20 image pairs acquired from volunteers as initial input (n_i_=40) and obtained 240 pieces of images that have directional information for both before (n_cb_=240) and after (n_ca_=240) acetic acid application. All detailed procedures with representative images for each step can be found in Multimedia Appendix 1.

### Feature Analysis and Selection

After the preprocessing of images, VIA features were analyzed to identify the images containing the suspicious lesions. To extract the abnormal features, we inspected RGB color intensity, values in extended color space, and Haralick’s texture features [33]. The application of the acetic acid on squamous epithelial areas coagulates the cellular protein and dehydrates the cytoplasm. Images of VIA– cervix, thus, generally showed light pink or very thin white appearances due to the reflection of light from the underlying stroma. On the other hand, VIA+ tissues that are rich in cellular proteins were presented with thick white features which blocked the colors of the stroma. Due to this reason, VIA+ incorporates larger, thick, white areas in images that would exhibit more green and blue intensities in color space [26,34]. From there, we computed the green-to-red and blue-to-red intensity ratio and found the separation between histogram distributions of pre– and post–acetic acid application images. This approach would properly quantify changes in green and blue intensities independent of device variation and level of illumination. The differences of histogram distribution of average (D_ave_), green-to-red (D_G/R_), blue-to-red (D_B/R_), and average histogram differences of green-to-red and blue-to-red (D_ave_) can then be defined as Standalone Equation 1, where I(mode) is intensity level or index at mode in histogram.


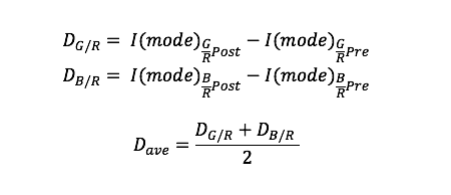


The standard deviations of the green and blue channels are another important extracted feature. Aceto-white features with higher green and blue channel intensities also exhibited higher standard deviations, which means more green and blue intensities are widely distributed in the histogram. Different color space was also utilized to find the features of VIA. Commission Internationale de l'Eclairage (CIE)*Lab color space was computed to achieve perceptual uniform color space, which is useful to quantitatively distinguish between the colors of an image. The great advantage of CIE*Lab is that it is independent of device and illumination [26].

In this study, the major color included on the cervix was defined by calculating the average of a* channels from each post-acetic application. Due to abnormal vascular formation, such as mosaicism and punctuation, visualization of the uneven surface of the ectocervix was another contrasting feature. Computation of Haralick’s texture feature using gray-level cooccurrence matrix (GLCM) may quantify the spatial variation of gray intensity values related to the texture of an image. GLCM measures the probability distributions of different combinations of pixel values. Utilizing the GLCM, several pieces of statistical information, such as contrast, correlation, energy, and homogeneity, can be derived quantitatively to exhibit the texture of the image [35,36]. Here, the GLCM was calculated at four different angles (0°, 45°, 90°, 135°) with an interpixel distance of 5 for the difference of the S channel (HSV color space) from pre– to post–acetic acid images. Different angles measure the features of interest in every direction. Therefore, all four GLCMs were summed before texture calculation.

In this study, we specifically utilized correlation statistics, which provide the extent of correlation between a pixel to its neighbor pixel over the whole image [35]. The correlation statistics (mean difference=0.248; *P*<.001) exhibited a significant difference in the VIA+/– images, yet other texture statistics had shown a very small difference, down to third and fourth decimal points in mean difference (contrast: *P*=.066; homogeneity: *P*=.308; energy: *P*=.249). Therefore, five VIA features were analyzed and selected as potentially useful for diagnostic classification: (1) Average difference of green/blue-to-red histogram index; (2) SD of green channel from post–acetic acid images; (3) SD of blue channel from post-acetic acid images; (4) average value of a* channel; and (5) correlation values of Haralick’s texture features from S channel information.

### Classification Training and Validation

Using selected features of VIA as predictors, we interpreted the tissue abnormality of a localized region for classification. Machine learning techniques have been widely used and successfully supervised for VIA classification [25,31]. In this work, we examined and selected an appropriate classifier by analyzing three different classifying methods. We performed holdout k-fold cross-validation (k=10), not only to optimize the hyperparameter to avoid overfitting/underfitting problems, but also to select the best performing model. Thus, we randomized the order of the images and used half (n_training_=120) for training classifiers. The other half of the data, an untrained image set (n_testing_=120), was used as the testing set after the optimization of classification models.

While in training, classifiers are validated using k-fold cross-validation (k=10) with histopathology labels as the ground truth. In this method, data is evenly divided into k subsamples. Other k-1 subsamples are then used as training datasets, and then held-out or excluded subsamples are used for validation. Algorithms were repeated k times, with each of the subsamples only utilized once, as the validating data and performance accuracy were calculated by averaging the results from each k-fold [37]. We designed k-nearest neighbors (KNN) with five neighbors based on Euclidian distance, support vector machine (SVM) with a fourth degree of polynomial kernel function (cost=3; gamma=2.2), and decision tree (DT) with a limit of a maximum of 20 nodes, based on Gini's diversity split criterion. These parameters, or hyperparameters, in each classifying method were optimized through the grid-search technique. All predictors were standardized using their corresponding weighted means and weighted standard deviations [28]. By using a validation result in training, receiver operating characteristic (ROC) curves with area under the curve (AUC), accuracy, sensitivity, and specificity of each trained classifier were computed and compared for selecting the best classifiers. Throughout the classifiers, each image was interpreted to either VIA+ or VIA–. The classification process is illustrated in Figure 3.

## Results

### Direct Evaluation of Smartphone-Based Endoscopic VIA

To determine the feasibility of the smartphone-based endoscope system for the VIA application, four physicians participated and reviewed the image sets (n=20). In these 20 cases, both clinically normal and low-grade squamous intraepithelial lesions (LSILs) were designated as “normal,” and high-grade squamous intraepithelial lesions (HSILs) was designated as “abnormal,” resulting in 8 normal and 12 abnormal cases for this study. The diagnostic performance assessed by pathologists is summarized in Table 1. Sensitivity ranged from 33.3-83.3%, and specificity was 100% for four physicians. Accuracy of the assessment ranged from 60.0%, with a Cohen kappa of 0.286 (*P*=.068), to 90.0%, with a Cohen kappa of 0.800 (*P*<.001). Overall, an average accuracy of 78%, with Cohen kappa of 0.571 (*P*<.001), was observed for the evaluation of the system.

**Table 1:** Sensitivity, specificity, Cohen kappa value, and *P* value for smartphone-based endoscopic VIA among all observers (n=20).

Physicians	Sensitivity, %	Specificity, %	Accuracy, %	Cohen Kappa	*P* value
Physician 1	75.0	100.0	85.0	0.706	.001
Physician 2	33.3	100.0	60.0	0.286	.068
Physician 3	83.3	100.0	90.0	0.800	<.001
Physician 4	58.3	100.0	75.0	0.528	.007
Average	62.5	100.0	77.5	0.571	<.001

### Result of Image Processing

**Figure 4:**
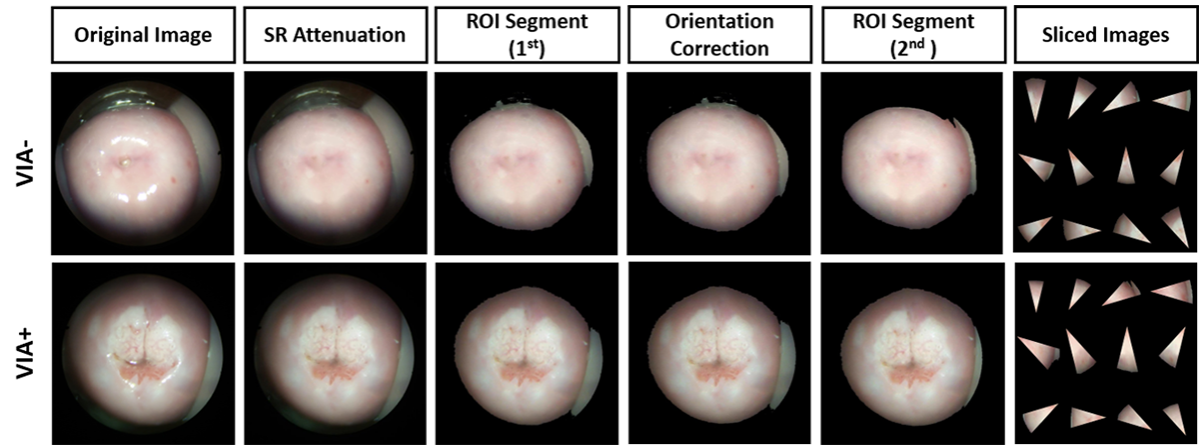
Result of image processing result. SR: speculum reflection; ROI: region of interest.

Figure 4 shows the representative images from each image processing step. Specular reflections on the surface of the cervix are removed while preserving visually natural or smooth features. All areas of specular reflection with saturated intensity are correctly localized for all 20 image pairs, including both pre– and post–acetic acid application images. The intensity threshold of 60% for specular reflection removal was effective, as the reflections on the ectocervix regions were identified clearly while minimally affecting general backgrounds. This was feasible because more reflection intensity was obtained in the focal plane compared to that of the unfocused regions. Next, the major cervical region was selected as the ROI and was segmented by applying the seventh and eighth bit-planes of the red channel image as the primary mask. Although most of the artifacts, such as unfocused background and speculum, were successfully eliminated, some images still contained portions of the vaginal wall. These results were likely found when the cervix and vaginal wall adjoined each other, or strong intensities appeared on the vaginal area by reflections. Through a semiautomated registration algorithm, images were centered, aligned, and rotated, as shown in the fourth column of Figure 4. Additional segmentation was conducted using overlapped regions of pre– and post–acetic acid application images as a secondary ROI mask. Furthermore, ROI images were sliced based on their clock positions as they were collected for histopathology. Each slice was then used to calculate color distribution histogram, CIE*Lab, and HSV-based Haralick’s texture features for VIA.

### Feature Extraction

**Figure 5:**
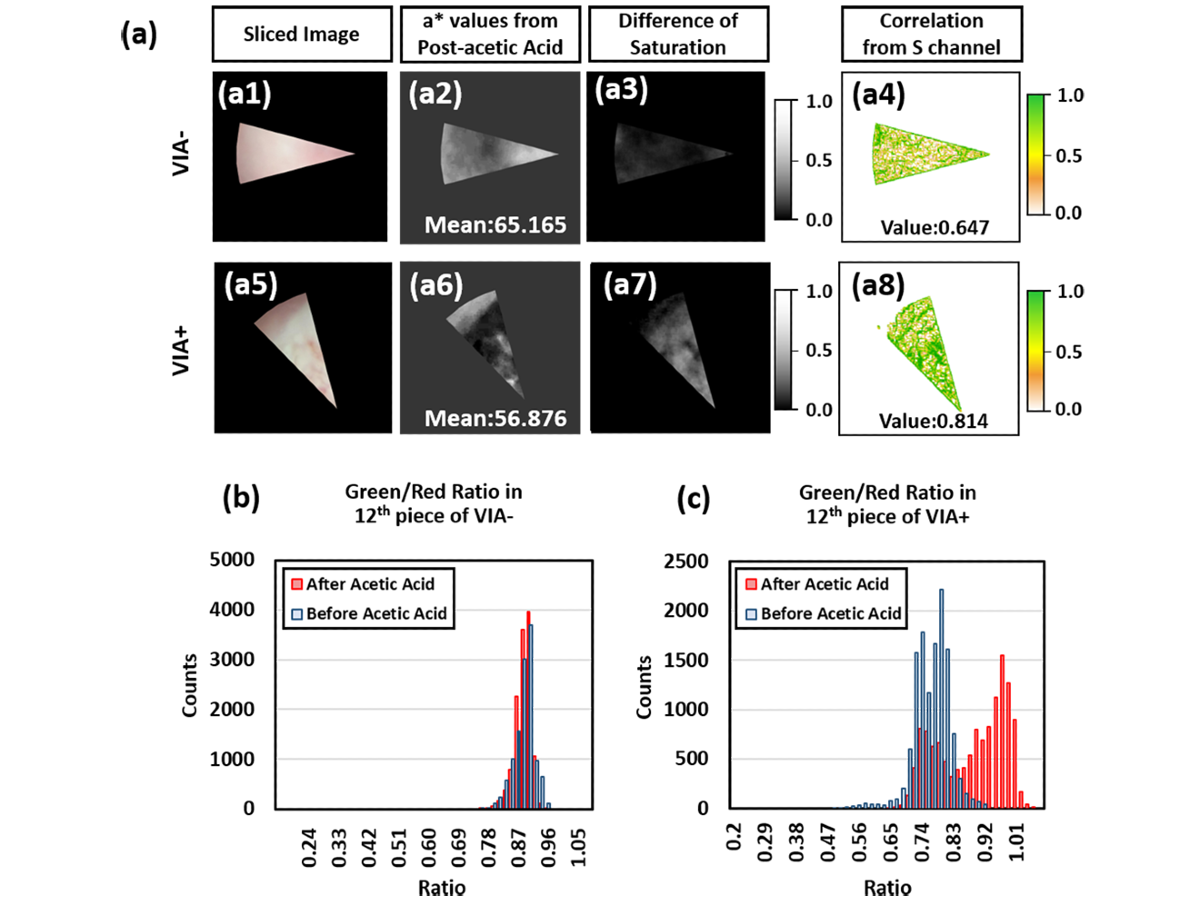
Result of feature extraction. (a) Extracted VIA features from sliced VIA-/VIA+ images, showing graphical information in different color spaces. In addition, correlation map was calculated form Haralick’s texture feature. (b,c) Representative histogram of green-to-red ratio in VIA-/VIA+. VIA: Visual inspection with acetic acid.

In Figure 5 (a1, a5), representative images of the sliced image are shown for VIA– and VIA+, respectively. Figure 5 (a2, a6) represents a* channel images using post–acetic acid images. Due to aceto-whitening areas, VIA+ showed significantly lower average values in the a* channel from CIE*Lab color space, which represents lower for green and higher for magenta colors. Primarily, lower values are localized in the area where aceto-white exists. In Figure 5 (a3, a7), the images of the difference of saturation channel from pre– to post–acetic acid application are shown. Higher values of saturation were obtained in aceto-whitening regions with VIA+ relative to those with VIA–, where no aceto-whitening regions exist. Using images in Figure 5 (a3, a7), correlation values were computed from Haralick’s texture feature and visualized in Figure 5 (a4,8). Contrary to expectation, higher correlation was found in VIA+ compared to VIA– (0-1, usually 1 for higher correlation); however, a significant difference was observed, with a good trend for distinguishing the feature.

**Figure 6:**
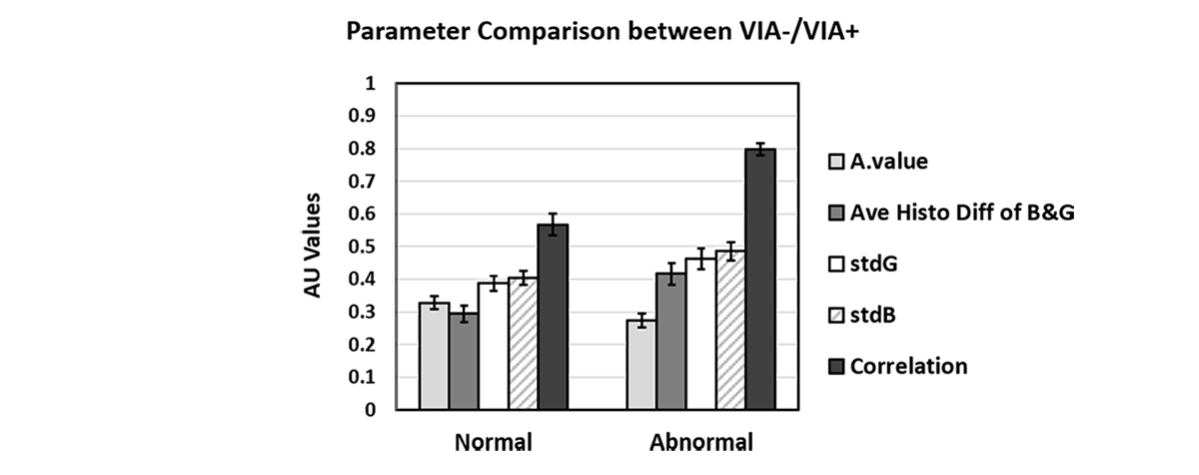
Summarization of selected features as predictors for classifying model. VIA: Visual inspection with acetic acid; AU: arbitrary unit.

Among the five different features, representative data for the green-to-red ratio were illustrated in Figure 5, part (b) and part (c). There was a relatively small effect from the acetic acid application observed in the green-to-red ratio in the VIA– images, and the histogram of the green-to-red ratio in VIA– shows little change between pre– and post–acetic acid application in terms of distribution and mode. However, the green-to-red ratio increased for VIA+ following application of acetic acid, as shown in the histogram in Figure 5, part (c). Following the application of acetic acid, the distribution of the green intensity histogram broadened along with an increase in intensity. Collecting all the statistical data, we derived the average difference of intensity level in green/blue-to-red ratio and the values of a* channels following application of acetic acid. The standard deviation of green and blue intensity and correlation of Haralick’s texture feature were also calculated from the images after the application of acetic acid. Figure 6 summarizes these features with all predictors in abnormal tissues having greater values than those from normal tissues, except for a* values.

### Classification Comparison

**Figure 7:**
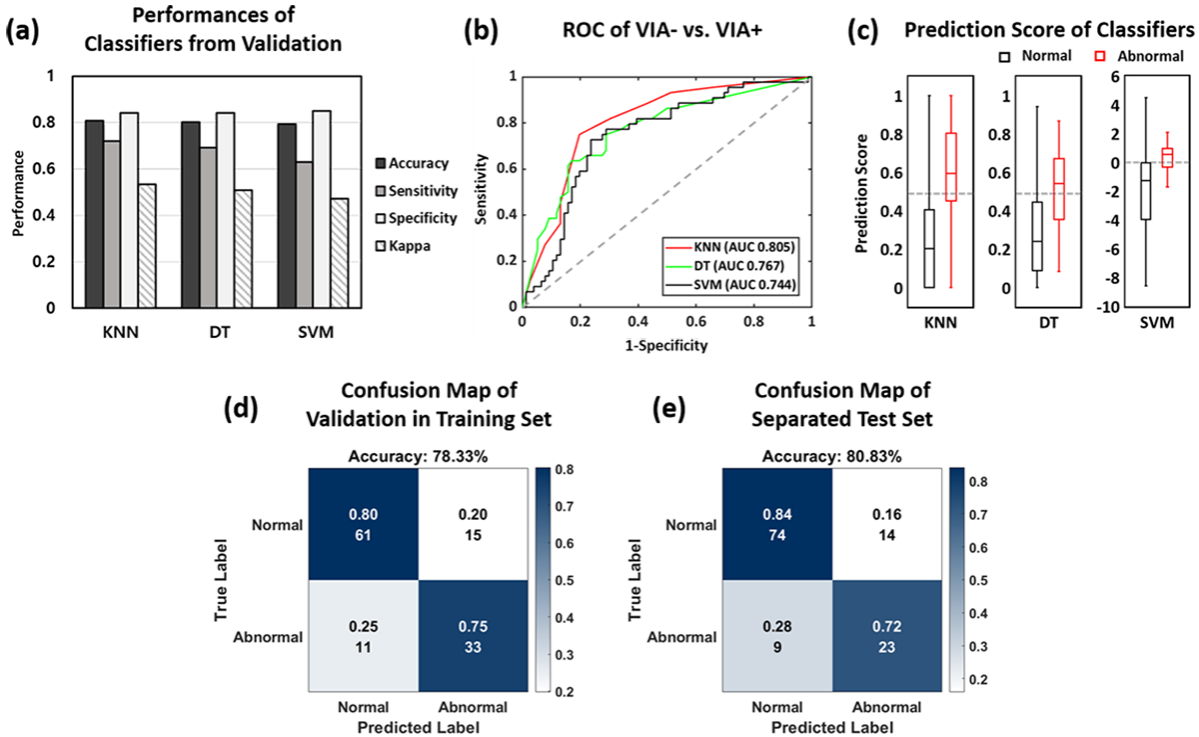
Comparison &amp; selection of classification model. (a) Performance of 10-fold cross validation in training set comparing the VIA results from each classifier. (b) ROC curves comparing the VIA+ performances. (c) Box plots of prediction scores for each classifying method. (d) Confusion map of validation in training set for KNN model. (e) Confusion map of separated test set for selected KNN model. VIA: visual inspection with acetic acid; ROC: receiver operating characteristic; KNN: k-nearest neighbors.

By using all the extracted values from 120 sliced image sets, three different types of machine learning classifiers were trained and analyzed. As shown in Figure 7, part (a), KNN yielded the best performance in 10-fold cross-validation. KNN had the best accuracy at 78.3%. with a sensitivity of 75.0%, a specificity of 80.3%, and a Cohen kappa of 0.5423 from 10-fold cross-validation. The second most accurate classifier was DT, with an accuracy of 75.8%, a sensitivity of 63.6%, specificity of 82.9%, and Cohen kappa of 0.4721. SVM’s performance was like DT but had the least accuracy for smartphone-based endoscopic VIA, with an accuracy of 74.2%, sensitivity of 72.7%, specificity of 75.0%, and Cohen kappa of 0.4618. In the ROC curve shown in Figure 7, part (b), KNN, DT, and SVM showed an AUC of 0.805, 0.767, and 0.744, respectively. Figure 7, part (c), illustrates the result of prediction scores from each classification on k-fold validation, indicating the probability of an image belonging to either the negative or positive class. From the box plot, separations of the scores of the abnormal and normal data were distinguishable in the KNN model when compared to that of DT and SVM. Most of the abnormal data were distributed over 0.5, but those of normal data were shown under 0.5 in the KNN and DT models. In the SVM model, normal and abnormal data were separated into positive and negative, respectively. As the best performing classifier in our study, the KNN model was selected to evaluate the diagnostic accuracy of VIA.

The confusion matrix for KNN in the validation and test sets is illustrated in Figure 7, parts (d) and (e). The trained KNN model produced similar accuracy for test data to that for validation data, with an accuracy of 80.8%. The sensitivity of 71.9% in the testing data was somewhat lower than that of validation data; however, the specificity of 84.1% was slightly higher than that of the training data.

### Analysis of the KNN Classification for Smartphone-Based Endoscopic VIA

**Figure 8:**
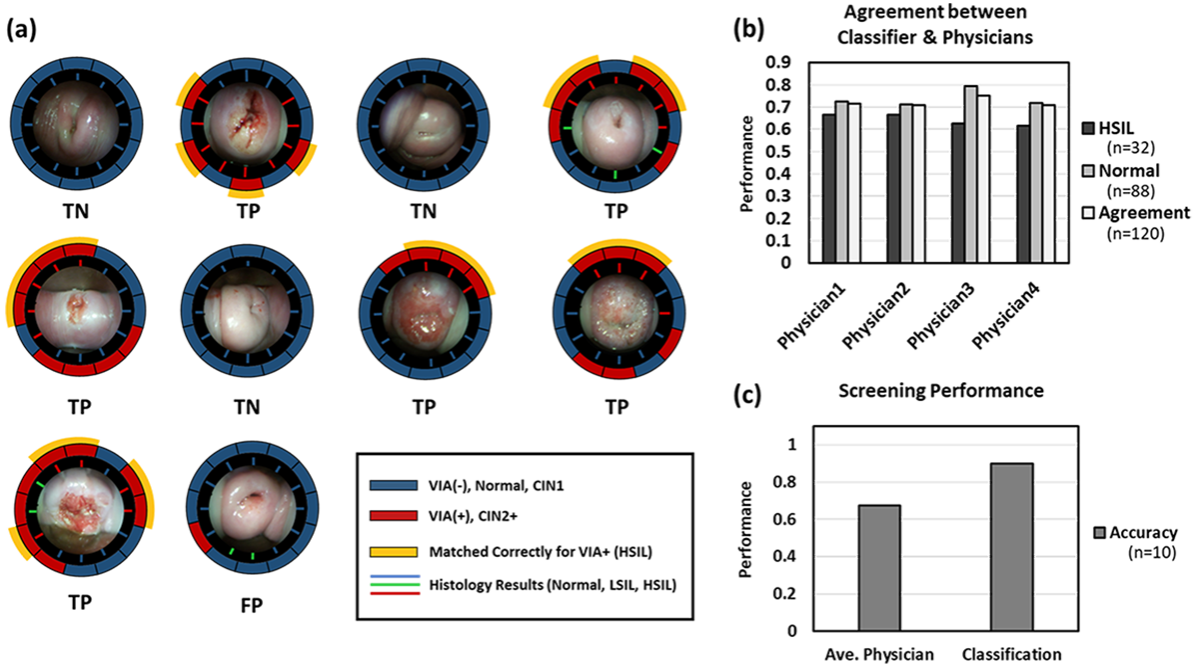
Analysis of KNN for smartphone-based endoscopic VIA. (a) Visualization of KNN classifying result for 10 patients with VIA+/VIA-. Lines inside of annulus ring indicate the results of the golden standard for each clock position. Annulus ring presents the result of KNN classification according to each clock position. Outer yellow mark represents the position where both the golden standard and prediction results are matched. (b) Graph presenting the agreement of KNN to each physician. (c) Graph comparing the screening performance for 10 patients interpreted by KNN and physicians. VIA: Visual inspection with acetic acid; KNN: k-nearest neighbors.

Figure 8 illustrates the result of the classification with smartphone-based endoscopic VIA images for ten patients that were used for the testing set. By using all the prediction results from the testing data, the locations of abnormal tissue were visualized as a segmented annulus outside of each endoscopic image. The red and blue segments of the annulus indicated VIA+ and VIA– at each clock position, respectively. Out of 120 slice images, 32 were positive and 88 were negative, according to the histopathology. As shown in Figure 8, part (a), all the results of the gold standard for each patient were denoted as colored lines inside the annulus and are aligned along each clock position as a line. Red, green, and blue lines denote abnormal tissues with HSIL (CIN2+), LSIL (CIN1), and normal diagnoses, respectively. When the KNN result was confirmed correctly as VIA+ by the gold standard, segments were labeled yellow on the perimeter of the annulus for each patient. A total of 23 sliced regions were matched correctly for VIA+.

Moreover, a patient was estimated to have an HSIL lesion if there was at least one VIA+ segment included among the 12 locations. Out of a total of 10 patients, six patients were predicted to have HSIL through KNN prediction. Among them, six patients were correctly estimated as VIA+ (true positive). Among the four patients confirmed as VIA–, KNN identified three patients correctly (true negative). However, there were no false negatives predicted by KNN for ten patients. Figure 8, part (b), shows the agreement between the classification algorithm and an individual physician’s interpretation for each clock position. The classification algorithm shows a moderate overall agreement ranging from 70.8-75.0%. For binary classifications of patients, the algorithm provides more accurate interpretations, as shown in Figure 8, part (c). The algorithm yielded an accuracy of 90%, and the average accuracy for the physician-achieved accuracy was 68% for ten patients.

## Discussion

### Principal Results

The findings of this study suggest that the novel smartphone-based approach for VIA with endoscopy and machine learning techniques could potentially be a useful tool for screening cervical cancer in low- and middle-income countries. A smartphone-based endoscope system is a simple and robust screening method that could be performed outside of the office without cost, mobility, and electricity limitations. Here, a moderate overall agreement (Cohen kappa=0.571) was achieved between the interpretations of the smartphone-based endoscopic cervicogram by physicians and the histopathologic results. With the support of machine learning and image processing techniques, the diagnostic performance was evaluated for classifying VIA– or VIA+ patients. We explored three different types of classifiers and selected the KNN algorithm for this study due to its classification performance, and its AUC of 0.805. Both hardware and machine-learned algorithms, based on the gold standard, overperformed compared to the conventional VIA. This was possible because our approach increased the sampling numbers of prediction for each patient compared to the gold standard. From the clinical point of view, finding abnormality in normal tissue is critical, especially in low-resource settings. The locations of abnormal tissue were identified and visualized at each clock position for each patient. With intuitive, perceptual color labels, VIA providers may easily understand where aceto-whitening has appeared and its degree of abnormality. Had our algorithm been used to determine the treatment pathway, the data would effectively assist the VIA providers with decision making.

To the best of our knowledge, this is the first smartphone-based endoscopic VIA work. A smartphone-based endoscopic approach provides some advantages over other smartphone-based VIA studies [14-20,38,39]. Many studies on diagnostic performances of smartphone VIAs have shown promising results, with sufficient accuracy [14-20]. These methods mostly utilized the tripod, or other hardware supports, to obtain pictures, which poses a challenge of limited space for maneuvering during the procedure. Recently, a portable colposcope device with a unique tampon form called the pocket colposcope was introduced with a high concordance to the clinical colposcope. Like our approach, a pocket colposcope enables us to capture images inside of the vagina. However, a pocket colposcope requires a wire-connection to a smartphone as an accessory, with limited hardware variation available [38,39]. Here, using endoscopic probes within a speculum offers noncontact, noninvasive imaging capability, minimizing the cross-contamination risk between patients. Our endoscopic probe enhances the resolution of the images and can achieve more details of mosaicism, punctuation, and aceto-white regions. This could substantially improve the diagnostic accuracy as well as quality control of VIA.

Moreover, various types of endoscopic probes bring flexibility to the system. A smartphone-based endoscope is based on off-the-shelf optics to magnify the images while reducing the signal-to-noise ratio by avoiding high digital zoom. A simple modification of optical components can enable additional functional imaging such as fluorescence, polarization, and multi-spectral imaging, and thus can further improve the visualization of the abnormal tissue [24,40-42]. Furthermore, manipulation of the endoscopic device inside of the vagina is very intuitive and easy compared to that of conventional approaches that are manipulated outside of the vagina. Thus, neither tripods nor stands are required for acquiring stable images, which in turn enhances the speed of the imaging session.

Image processing algorithms have been developed to minimize external sources of error, such as deviations of illumination power, imaging position, variations of devices, specular reflections, and ROI of the cervix. Our method of ROI segmentation is especially unique, as it uses bit-plane separation and finding overlapped regions to eliminate insufficient information in smartphone-based endoscopic cervicograms. VIA features were mostly extracted based on a statistical calculation of histograms rather than just acquiring the quantitative pixel information from the raw images. This may minimize dependencies on the imaging environment and make image calculation confined to each image, but still afford similar values of computation results when finding features. Extending the RGB images to other color space also provides benefits for calculation. Saturation values from HSV color space afford the extent of purity of the hue data, which results in more intuitive perceptual values between pre– and post–acetic acid application.

Moreover, the saturation value is separated from the brightness value; thus, it is illumination invariant. Empirically, using images of the difference between pre– and post–acetic acid application in the saturation channel for GLCM showed a higher significant difference *(P<.*001) in correlation values between VIA– and VIA+. CIE*Lab color space is also very useful for finding device- or illumination-independent quantitative color measurements. CIE*Lab also separates the luminance component (L*) from the color, which makes color information less sensitive to illumination [28].

### Limitations and Future Works

Even though our novel CC screening technique using smartphone-based endoscopic VIA and machine learning demonstrated a positive outcome as a pilot study, much improvement is needed before it can reach its practical potential. First, the accuracy of our algorithm classifying the patients was calculated using a small number of samples. No false-negative data was predicted within a small number of samples. We believe increasing sampling size could additively provide a greater number of sliced data for training and testing classifiers, which could lead to more new cases that eventually lead to much more reliable classification results. Therefore, the overall estimation could be different with a larger data set; however, it will still provide better performance compared to subjective estimation from physicians. Second, our approach for ROI segmentation aimed to precisely distinguish the ectocervix area from the images; however, there are still some portions of vaginal walls left that led to misclassification.

Moreover, the location and the area of the cervix are substantially different in each image. For this reason, the image processing algorithm for registering images of pre– and post–acetic acid application was done by manually providing positions of the center and distal ends of the cervical os. Through image standardization by providing the visual guides in software, deviation of positioning and working distance can be minimized and further improve the ROI segmentation performance in an automated processing algorithm. Lastly, our image processing–based classification algorithm was performed on a computer, limiting practical uses in developing countries in its current form. Due to the computational intensity, the current algorithm cannot be operated as a standalone in a smartphone. Recently, Android-based machine learning, as well as image processing techniques, have been introduced in various fields, using SVM, KNN, DT, and Deep Convolutional Neural Networks. As these functions are now available on mobile phones [43-46], we will embed these Android-based machine learning techniques to implement our algorithm on a smartphone alone.

### Conclusions

In conclusion, we explored the use of smartphone-based endoscopic VIA and predicted a cervicogram as normal/abnormal using machine learning techniques. Herein, histopathology results, acquired at each clock position on the excised cervical tissue, were provided as ground truth for classifications. VIA features were then extracted after image processing and utilized for training the classifiers. Overall, 120 sliced images obtained from the cervicogram at each clock position were utilized, and three classifiers, such as KNN, DT, and SVM, were compared. Approaches using KNN showed the best performance from holdout 10-fold cross-validation in the training set, with an accuracy of 78.3%, AUC of 0.807, a specificity of 80.3%, and sensitivity of 75.0%. To validate the trained model, we used the other 120 sliced images, achieving an accuracy of 80.8%, specificity of 84.1%, and sensitivity of 71.9%. Prediction values were visualized with intuitive color labels, indicating the normal/abnormal tissue at each clock position for each patient. Calculating the overlapped abnormal tissues between the gold standard and predicted values, our KNN model for classifying VIA–/VIA+ patients overperformed the interpretation results by physicians. Taken together, these results suggest that the smartphone-based endoscopic VIA and analysis based on the machine learning techniques would be a valuable tool for screening VIA in low-resource settings. Moreover, this approach can potentially minimize human subjectivity and be particularly useful in areas where experts or teleconsultations are unavailable.

## Appendix

Multimedia Appendix 1 Block diagram of image processing and classification. (a) Pre-image processing method, (b) Feature analysis and classification.

## Abbreviations

3D: three-dimensional
AU: arbitrary units
AUC: area under the curve
CC: cervical cancer
CIE: Commission Internationale de l'Eclairage
CIN: cervical intraepithelial neoplasia
CV: cross-validation
DT: Decision tree
GLCM: gray-level cooccurrence matrix
HSIL: high-grade squamous intraepithelial lesion
HSV: Hue, Saturation, Value
KNN: k-nearest neighbors
LEEP: loop electrosurgical excision procedure
LSIL: low-grade squamous intraepithelial lesion
RGB: red, green, blue
ROC: receiver operating characteristic
ROI: region of interest
SVM: support vector machine
USAF: Unites States air force
VIA: visual inspection with acetic acid
