# Supplementary figures and images for: Quantitative Screening of Cervical Cancers for Low-Resource Settings: Pilot Study of Smartphone-Based Endoscopic Visual Inspection After Acetic Acid Using Machine Learning Techniques
Source: JMIR Mhealth Uhealth. 2020 Mar 11;8(3):e16467. doi: 10.2196/16467 (PMC7097827; doi:10.2196/16467)

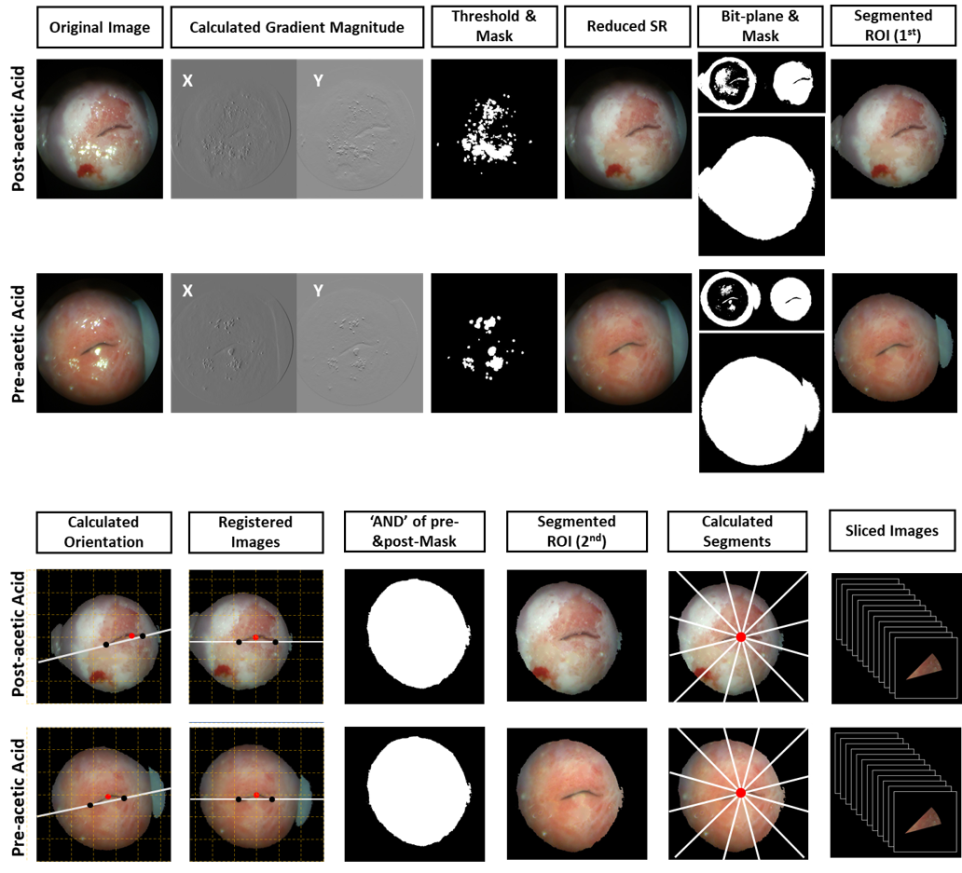

Supplement: Multimedia Appendix 1 [file mhealth_v8i3e16467_app1.png]
